# Unveiling Metabolic Vulnerability and Plasticity of Human Osteosarcoma Stem and Differentiated Cells to Improve Cancer Therapy

**DOI:** 10.3390/biomedicines10010028

**Published:** 2021-12-23

**Authors:** Gerardo Della Sala, Consiglia Pacelli, Francesca Agriesti, Ilaria Laurenzana, Francesco Tucci, Mirko Tamma, Nazzareno Capitanio, Claudia Piccoli

**Affiliations:** 1 Department of Marine Biotechnology, Stazione Zoologica Anton Dohrn, Villa Comunale, 80125 Naples, Italy; gerardo.dellasala@szn.it; 2 Laboratory of Pre-Clinical and Translational Research, IRCCS-CROB, Referral Cancer Center of Basilicata, 85028 Rionero in Vulture, Italy; ilaria.laurenzana@crob.it; 3 Department of Clinical and Experimental Medicine, University of Foggia, 71122 Foggia, Italy; consiglia.pacelli@unifg.it (C.P.); francesca.agriesti@unifg.it (F.A.); francesco.tucci@unimi.it (F.T.); mirko_tamma555271@unifg.it (M.T.)

**Keywords:** oncometabolism, cancer stem cell, metabolic therapy, mitochondria, cisplatin, osteosarcoma, metabolic flux analysis, impedentiometry

## Abstract

Defining the metabolic phenotypes of cancer-initiating cells or cancer stem cells and of their differentiated counterparts might provide fundamental knowledge for improving or developing more effective therapies. In this context we extensively characterized the metabolic profiles of two osteosarcoma-derived cell lines, the 3AB-OS cancer stem cells and the parental MG-63 cells. To this aim Seahorse methodology-based metabolic flux analysis under a variety of conditions complemented with real time monitoring of cell growth by impedentiometric technique and confocal imaging were carried out. The results attained by selective substrate deprivation or metabolic pathway inhibition clearly show reliance of 3AB-OS on glycolysis and of MG-63 on glutamine oxidation. Treatment of the osteosarcoma cell lines with cisplatin resulted in additive inhibitory effects in MG-63 cells depleted of glutamine whereas it antagonized under selective withdrawal of glucose in 3AB-OS cells thereby manifesting a paradoxical pro-survival, cell-cycle arrest in S phase and antioxidant outcome. All together the results of this study highlight that the efficacy of specific metabolite starvation combined with chemotherapeutic drugs depends on the cancer compartment and suggest cautions in using it as a generalizable curative strategy.

## 1. Introduction

Osteosarcoma (OS) is the most common among primary malignant bone tumors, with widespread occurrence in children and adolescents aged 10–25 years [1]. If chemotherapy and surgical resection have improved clinical outcome of patients with localized tumors, most patients suffer relapses and the high metastatic potential of OS contributes to poor prognosis. Current chemotherapy regimen includes cisplatin, methotrexate, and doxorubicin [2]. However, owing to the frequent onset of chemoresistance as well as the lack of an effective second-line chemotherapy in patients with recurrent/metastatic OS [3], identification of new therapeutic strategies is mandatory.

Novel insights into the different metabolic addictions of tumors have provided interesting clues to exploit altered metabolism in cancer cells for innovative therapeutic approaches, some of which are currently under evaluation in clinical trials [4,5]. Since Otto Warburg reported that cancer cells relied on aerobic glycolysis (Warburg effect) and featured defective mitochondrial respiration in 1924 [6], deciphering the complex metabolic network in cancer cells has made great strides and has become one of the most attractive research areas. It is now currently acknowledged that the “Warburg effect” is not a general feature of all cancers and that glycolysis and oxidative phosphorylation (OxPhos) are not mutually exclusive events, as contributing differently to ATP production in response to environmental changes (e.g., nutrient and oxygen supply) [7]. Moreover, in contrast with the Warburg theory, cancer cells often display functional mitochondria, which turn out to play a key role in cell survival and proliferation as well as to confer resistance to conventional chemotherapy [8,9,10]. A bulk of studies reported that oncogenic signaling and tumor microenvironment drive an energetic reprogramming involving metabolic pathways, such as glutaminolysis, fatty acid oxidation and redox homeostasis [11,12]. Therefore, targeting glycolysis and/or mitochondrial metabolism could provide a promising Achilles’ heel to kill cancer cells and integrate chemotherapy into combined modality treatments. Most of treatments currently included in clinical settings are based upon the stochastic model. According to this model, cancer cells derive from a distinct set of progenitor cells which undergo gene mutations to gain a highly proliferative state and develop metastatic capability. However, an increasing body of evidence has shed light on the existence of a sub-population of tumor-initiating cells, namely cancer stem cells (CSCs), which are able to self-renew and generate progenitor tumor cells (hierarchical model) [13,14]. As being typically quiescent within a tumor mass, CSCs have turned out to be resistant to traditional drugs targeting high proliferating cells and, consequently, are responsible for tumor recurrence and relapse. Bioenergetics of the CSCs pool rely primarily on glycolysis, although multifactorial causes may induce plasticity, thus accounting for a unique metabolic phenotype far different from that of differentiated cancer cells. Interestingly, even CSCs localization within a tumor mass may affect their metabolic dependency. In normoxic areas of the tumor, CSCs have been shown to use either glycolysis or OxPhos for ATP and NADH production, while in hypoxic regions CSCs upregulate glycolytic enzymes. When embedded in the metastatic niche, CSCs often exhibit increased capability to oxidize extracellular substrates (pyruvate, lactate, glutamine, glutamate, alanine, or ketone bodies), whereas, during nutrient starvation, alternative fuel sources may arise from autophagy [15]. Advances in therapeutic intervention have highlighted the importance of killing CSCs for complete eradication of tumors [16].

In this scenario, 3AB-OS osteosarcoma mesenchymal stem cell line can be regarded as an attractive model to investigate metabolic dependence and flexibility for the search of valuable targets for anticancer therapy. 3AB-OS cells, which have been isolated from the parental MG-63 cell line after long-term exposure to 3-aminobenzamide, express many genes essential for maintaining stemness, and are able to retain their morphological and antigenic profiles even after 200 passages [17,18]. In addition, Di Fiore et al. have demonstrated the high tumorigenic potential of 3AB-OS, reproducing features of the human osteosarcoma in vivo by xenograft implantation of 3AB-OS CSCs in mice [19].

Comparative metabolic analysis highlighted 3AB-OS CSCs and MG-63 parental cells to possess two distinct metabolic phenotypes [20]. Herein, our study lays bare metabolic vulnerabilities of osteosarcoma cell lines for validating metabolism-modulating approaches as a mean of killing cancer cells. Intersections between metabolic modulation and cancer cell growth are investigated either through energy substrate deprivation or inhibition of specific pathways by using antimetabolites. Finally, our report aims to provide the rationale for a combination therapy which integrates cisplatin chemotherapy with tailored manipulation of energetic pathways, to target both stem and differentiated cells. In this frame, an unexpected finding was the onset of antagonist effects of energy substrate withdrawal and cisplatin administration on 3AB-OS proliferation, thereby leading to promotion of CSCs maintenance. Reprograming and regulation of mitochondrial functionality are proposed as mechanisms underlying the observed antagonist effects in CSCs.

## 2. Materials and Methods

### 2.1. Cell Culture

MG-63 cells were purchased from American Type Culture Collection (ATCC, Manassas, VA, USA) and 3AB-OS cells were gently provided by Prof. Luigi Del Vecchio (Department of Molecular Medicine and Medical Biotechnologies, University of Naples Federico II). For normal growth condition, i.e., high glucose, both cell lines were cultured at 37 °C in a 5% CO_2_ humidified atmosphere in complete DMEM medium containing 25 mM glucose, supplemented with 10% fetal bovine serum, penicillin–streptomycin (100 U/mL), 2 mM glutamine (all these products were purchased from Thermo Fisher Scientific, Waltham, MA, USA). To evaluate cell response during glutamine shortage, cells were grown in complete high glucose DMEM medium, supplemented with different glutamine concentrations as indicated. Alternatively, to evaluate cell response in glucose-limiting conditions, cells were grown in complete DMEM medium, supplemented with 2 mM glutamine and different concentrations of glucose as indicated. Cisplatin and bafilomycin A were purchased from Sigma Aldrich (St. Louis, MO, USA); compounds were added to medium for the reported doses and time periods. Cell morphology was observed at inverted optical microscope (Axio Vert A1, Zeiss, Oberkochen, Germany).

### 2.2. Metabolic Flux Analysis

For real-time monitoring of the oxygen consumption rate (OCR) and extra-cellular acidification rate (ECAR) in adherent MG-63 and 3AB-OS cells, we used an XF96 extracellular flux analyzer (Seahorse Bioscience, Billerica, MA, USA). OCR was measured under basal conditions as well as after sequential injection of oligomycin (1 μM), FCCP (1 μM), and rotenone + antimycin A (1 μM + 1 μM) to evaluate the ATP-linked respiration, the maximal respiratory capacity, and the non-mitochondrial oxygen consumption, respectively. Glycolytic activity was monitored by measuring ECAR after sequential injection of 10 mM glucose, 1 μM oligomycin, and 100 mM 2-deoxyglucose, to assess basal glycolysis, maximum glycolytic capacity, and non-glycolytic acidification, respectively.

For measuring mitochondrial usage of glucose, glutamine, and long-chain fatty acids (i.e., palmitate), the Seahorse XF Mito Fuel Flex Test was performed, using the XF96 extracellular flux analyzer (Seahorse Bioscience, Billerica, MA, USA), according to the manufacturer’s protocol. Concisely, mitochondrial dependency on and flexibility for each substrate is determined by measuring the decrease in oxygen consumption rate upon exposure to one or more inhibitors of the relevant fuel oxidation pathways. Particularly, UK5099 (2 µM), BPTES (3 µM) and Etomoxir (4 µM) (Agilent Technologies, Milan, Italy) were used as inhibitors of the glucose, glutamine, and long-chain fatty acid oxidation pathways, respectively. The OCR and ECAR values were normalized to protein content in each well, determined using BCA assay (Thermo Scientific, Waltham, MA, USA).

### 2.3. Mitochondrial DNA Quantification

Mitochondrial DNA quantification was assessed as previously described [21]. Copy number of mtDNA, relative to nuclear DNA copy number, was determined by amplification of mitochondrial tRNA-Leu (UUR) and nuclear-encoded β2m (beta-2-microglobulin) genes. Total DNA extraction was performed by using the QIAmp DNA mini kit (Qiagen, Hilden, Germany), following the manufacturer’s protocol. 6 ng of total DNA were amplified by PCR in a LightCycler^®^ 480 real-time PCR Instrument (Roche Diagnostic, Basel, Switzerland) with the Light Cycler^®^ 480SYBR Green I Master (Roche Diagnostics, Basel, Switzerland), using Human β2m forward (5′-TGCTGTCTCCATGTTTGATGTATCT-3′) and β2m reverse (5′-TCTCTGCTCCCC ACCTCTAAGT -3′) or tRNA-Leu(UUR) forward (5′-CACCCAAGAACAGGGTTTGT—3′) and tRNA-Leu(UUR) reverse (5′-TGGCCATGGGTATGTTGTTA-3′) primer pairs. Realtime PCR reactions were carried out under the following conditions: 50 °C for 2 min, 95 °C for 10 min, followed by 40 cycles of 95 °C for 15 s and 62 °C for 60 s. Mitochondrial DNA quantification was calculated using the following equations: ΔCT = (nucDNA CT-mtDNA CT); relative mitochondrial DNA content = 2 × 2^ΔC^.

### 2.4. Live Cell Imaging of mtΔΨ, ROS, and Mitochondrial Morphology

Cells cultured at low density on fibronectin-coated 35-mm glass-bottom dishes (Eppendorf, Hamburg, Germany) were incubated for 20 min at 37 °C with (a) 2 μM TMRE (Tetramethylrhodamine ethyl ester perchlorate) or 0.5 μM MitoTracker Red to monitor mtΔΨ, (b) 10 μM DCF (dichlorofluorescein) to evaluate ROS production. Fluorescent dyes were purchased from Molecular Probes (Eugene, OR, USA). Stained cells were washed with PBS and examined using a Leica TCS SP8 confocal laser scanning microscope (Leica Microsystems, Wetzlar, Germany). Acquisition, storage, and data analysis were performed using the Leica Application Suite integrated software (LAS-X, Leica Microsystems, Wetzlar, Germany) and the ImageJ software (Available online: https://imagej.nih.gov/ij/download.html (accessed on 10 April 2020)).

### 2.5. Real-Time Monitoring of Cell Growth by the xCELLigence System

The xCELLigence System Real-Time Cell Analyzer (ACEA Biosciences, San Diego, CA, USA) was used for monitoring cell proliferation, as previously reported [22]. As opposed to traditional dye-based analysis, the xCELLigence RTCA is advantageous in that it (a) provides a real-time and kinetic readout, (b) allows non-invasive, physiological measurement of cell proliferation, (c) allows to monitor cell density, morphology and fitness, thereby ensuring more accurate and reproducible data. MG-63 and 3AB-OS cells were seeded at a cell density of 4000 cells/well in 16-well E-plates, connected to the RTCA platform placed into a cell incubator at 37 °C and 5% CO_2_. In general, when cells reached log growth phase approximately 24 h after seeding, medium was removed, and cells were treated as indicated. Only in the case of cell viability assays described in Section 3.5, cells were exposed to specific pretreatments for 48 h, and then treated as reported. Cell proliferation was monitored every 30 min for a period of up to 96 h (up to 120 h for cell viability assays in Section 3.5), measuring alterations of electrical impedance which were translated into a dimensionless parameter, namely cell index (CI). CI was normalized just before treatment and converted into normalized cell index (NCI), by the RTCA-integrated software (Version 2.0, ACEA Biosciences, San Diego, CA, USA). Normalized cell index was calculated as follows: NCI = CI_end of treatment_/CI_normalization time_.

### 2.6. Apoptosis Assay and Cell-Cycle Analysis

After exposure to glucose shortage and/or treatment with different concentrations (5 and 10 µM) of cisplatin for 24, 48 and 72 h, 3AB-OS cells were stained with annexin-V-fluorescein isothiocyanate (FITC) and propidium iodide, using the FITC Annexin V Apoptosis Detection kit I (Becton Dickinson, BD, Franklin, NJ, USA) for flow cytometric detection of live, apoptotic and necrotic cells. Samples were prepared according to manufacturer’s protocol. For cell cycle analysis of 3AB-OS cells incubated in high (25 mM)/low (1 mM) glucose conditions with or without cisplatin (5 and 10 µM) for 24, 48 and 72 h, osteosarcoma stem cells were permeabilized with 70% cold ethanol for 1 h and stained for 30 min with a solution containing 50 µg/mL propidium iodide (Sigma Aldrich, St. Louis, MO, USA) and 10 µg/mL RNase A (EuroClone S.p.a., Pero, Milan, Italy) in calcium and magnesium-free PBS. All samples were acquired by NAVIOS flow cytometer and analysed by Kaluza software (Beckman Coulter, Brea, CA, USA). 10,000 events were acquired for each sample.

### 2.7. Western Blotting Analysis

Aliquots of cell lysates, containing 40 μg of proteins, were subjected to SDS polyacrylamide gel electrophoresis and proteins were blotted on a polyvinylidene difluoride (PVDF) membrane using a Trans Blot Turbo Transfer System (Bio-Rad Laboratories, Hercules, CA, USA). PVDF membranes (Bio-Rad Laboratories, Hercules, CA, USA) were probed with the following primary antibodies SIRT3, SIRT1, LC3B, Cyclin B1, and p53 (1:1000, Cell Signaling Technology, Danvers, MA, USA); p21/WAF1/Cip1 clone CP74, Cyclin E, and Cyclin D1/D2 (1:1000, EDM Millipore Temecula, CA, USA); Cdk1/2 (1:1000, Santa Cruz Biotechnology, Santa Cruz, CA, USA); p27 (1:1000, Abcam, Cambridge, MA, USA). After incubation with a correspondingly suited horseradish peroxidase-conjugated secondary antibody (1:2500, Cell Signaling Technology, Danvers, MA, USA), chemiluminescent signals were developed using the Clarity Western ECL Substrate (Bio-Rad Laboratories, Hercules, CA, USA) and captured with the ChemiDoc imaging system XRS + (Bio-Rad Laboratories, Hercules, CA, USA). To read band intensity of the target proteins, images were analyzed using the Image Lab software (version 6.0, Bio-Rad, Hercules, CA, USA). The intensity of the relevant bands was normalized to the β-actin signal (1:5000, Sigma Aldrich, St. Louis, MO, USA).

### 2.8. Statistical Analysis

Data are reported as the mean (±standard deviation, SD or ±standard error, SEM) of at least three independent experiments. The GraphPad Prism Software Version 5 (GraphPad Software Inc., San Diego, CA, USA) was used for data statistical analysis. Student’s *t*-test and one-way analysis of variance (ANOVA) were used to compare means between groups. Bonferroni or Dunnett tests were used for post hoc analysis in ANOVA for multiple comparisons. Differences were considered statistically different if *p*-values < 0.05.

## 3. Results

### 3.1. Metabolic Profiling of 3AB-OS Cancer Stem Cells and MG-63 Differentiated Cancer Cells

**Figure 1:**
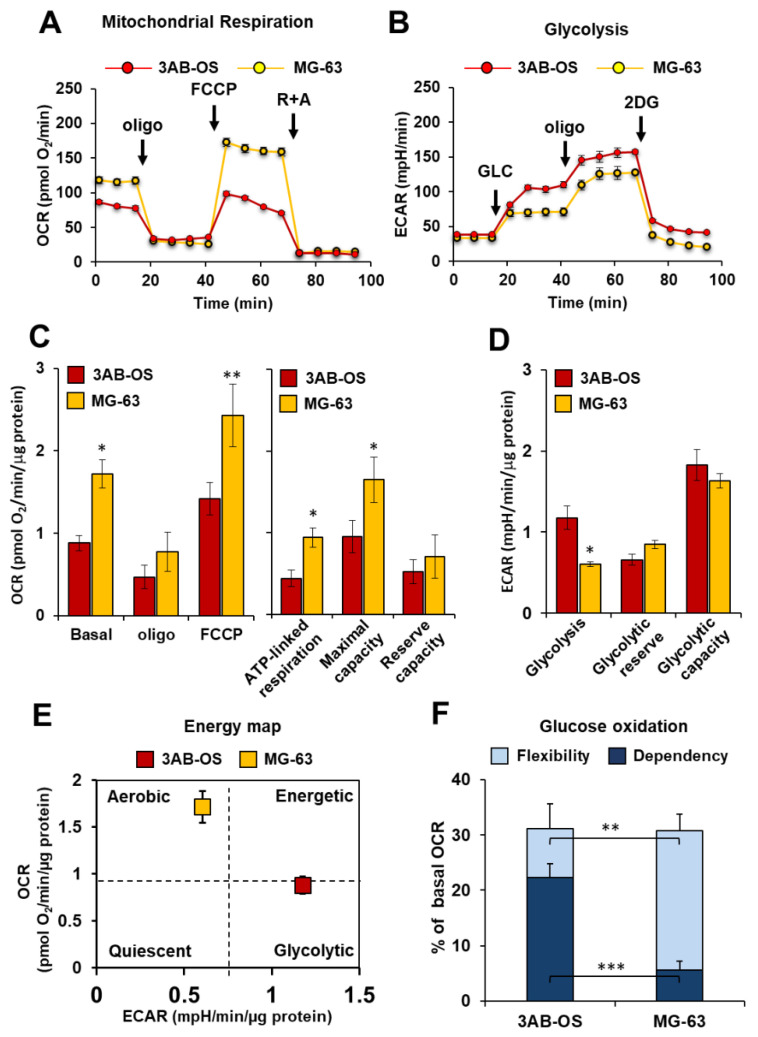
Comparative metabolic flux analysis of 3AB-OS and MG-63 cells, by using the Seahorse XFe96 Analyzer. (**A**) Representative profile of mitochondrial respiration stress test assay of 3AB-OS and MG-63 cells in basal condition, in presence of the ATP-synthase inhibitor oligomycin and after addition of the mitochondrial uncoupler FCCP. (**B**) Representative profile of glycolytic activity stress assay of MG-63 and 3AB-OS cells in presence of saturating amount of glucose (glycolysis), in presence of the glycolysis inhibitor 2-deoxyglucose (reserve), and after addition of the ATP-synthase inhibitor oligomycin (capacity). (**C**) Statistical analysis of the OCRs measured as in (**A**). Left panel: histograms showing basal OCR, OCR in the presence of oligomycin, OCR in the presence of FCCP; right panel: histograms showing ATP-linked respiration, maximal respiratory capacity and reserve capacity computed as described in Materials and Methods. The values are means  ±  SD of four independent experiments. * *p*  <  0.05; ** *p*  <  0.01. (**D**) Statistical analysis of the ECARs measured as in (**B**). The histograms show basal glycolysis, glycolytic reserve and glycolytic capacity as described in Materials and Methods. The values are means  ±  SD of four independent experiments. * *p*  <  0.05. (**E**) Energy map was obtained plotting basal OCR versus basal ECAR of 3AB-OS and MG-63 cells. (**F**) Evaluation of mitochondrial glucose usage in 3AB-OS and MG-63 cells, expressed as percentage of basal OCR. Glucose dependency indicates cell reliance on the glucose oxidation pathway to maintain basal respiration. Metabolic flexibility to glucose is the ability of cells to compensate for the inhibition of the glucose oxidation pathway by oxidation of alternative substrates to fuel mitochondrial OxPhos. The values are means  ±  SD of three independent experiments. ** *p*  <  0.01; *** *p*  <  0.001. Abbreviations: *oligo*, oligomycin; *FCCP*, carbonyl cyanide 4-(trifluoromethoxy)phenylhydrazone; *R*, rotenone; *A*, antimycin A; *GLC*, glucose; *2DG*, 2-deoxy-D-glucose; *OCR*, oxygen consumption rate; *ECAR*, extracellular acidification rate.

Metabolic flux analysis performed by Seahorse methodology showed in 3AB-OS CSCs decreased mitochondrial respiratory functions compared to differentiated osteosarcoma cells MG-63 (Figure 1A). In particular, 3AB-OS cells unveiled (a) lower ATP turnover, calculated as the oxygen consumption rate (OCR) used for mitochondrial ATP synthesis and (b) reduced maximal mitochondrial capacity, after treatment with the mitochondrial oxidative phosphorylation uncoupler FCCP (Figure 1C). As regards the reserve capacity, i.e., the difference between maximal and basal OCR, no significant difference was observed between the two cell lines as MG-63 cells are very close to their maximum OCR under basal conditions.

In parallel with the reduced mitochondrial activity, 3AB-OS CSCs showed a markedly glycolytic phenotype as underlined by increased extracellular acidification rate (ECAR) largely due to the higher rate of lactate production as compared to MG-63 cells (Figure 1B–E). These results confirmed what previously reported [20].

Moreover, mitochondrial glucose dependency as well as metabolic flexibility to glucose were evaluated within both cell lines. For quantitative assessment of mitochondrial glucose usage in live cells the Mito Fuel Flex Test was performed, by measuring OCR in presence/absence of the mitochondrial pyruvate carrier inhibitor UK5099. As compared to MG-63 cells, 3AB-OS CSCs exhibited (a) a greater dependency on pyruvate oxidation pathway and (b) a lower flexibility in compensating for the inhibited pathway by oxidation of alternative substrates to fuel mitochondrial respiration (Figure 1F).

**Figure 2:**
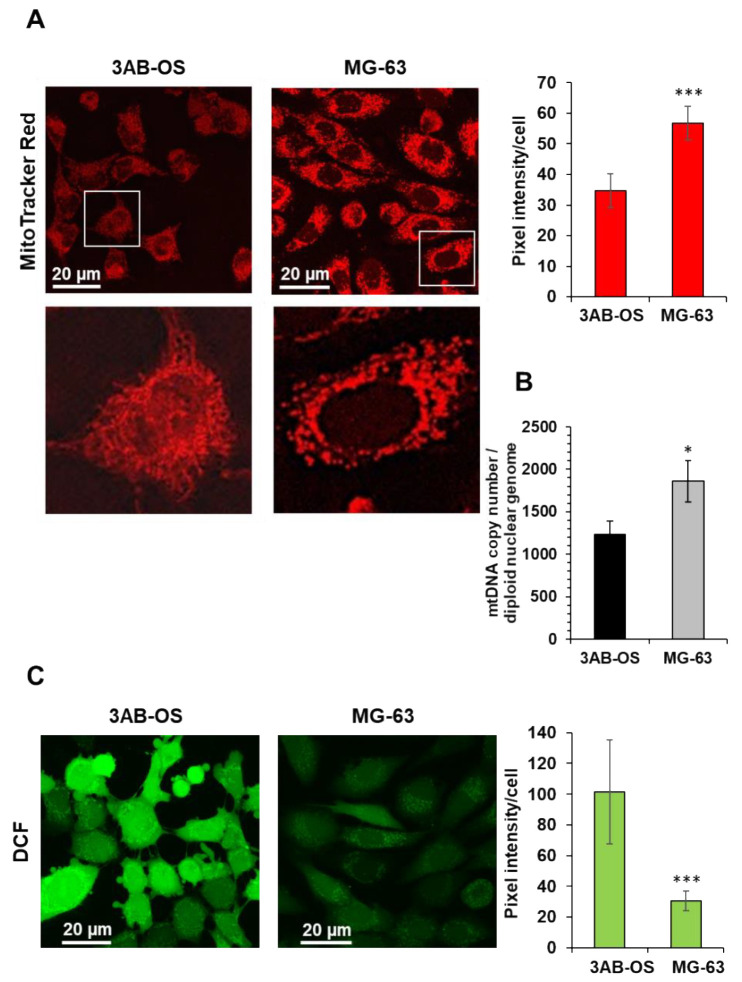
(**A**) Laser scanning confocal microscopy (LSCM) imaging of mitochondrial morpho-functional parameters in 3AB-OS and MG-63 cells by the fluorescent probe MitoTracker Red. Left panels show representative images and digital magnifications of the squared details. The histogram on the right shows statistical analysis of the MitoTracker pixel intensity per cell; values are means ±  SD of three independent biological replicates; *** *p*  <  0.001. Ten randomly chosen optical fields/sample each containing about 15 cells were analyzed by ImageJ. (**B**) mtDNA copy number of 3AB-OS and MG-63 cells assessed by q-RT-PCR; the bar histogram shows values normalized to the nuclear DNA. The values are means  ±  SD of three independent experiments; * *p*  <  0.01. (**C**) LSCM imaging of cells treated with the reactive oxidant species probe dichlorodihydrofluorescein (DCF). Left panels show representative images. The histogram on the right shows statistical analysis of the DCF pixel intensity per cell, *** *p*  <  0.001; values are means ±  SD of three independent biological replicates analyzed as in (**A**).

The reduced OxPhos flux in 3AB-OS CSCs correlated with the reduced mitochondrial membrane potential (Figure 2A) and the lower mtDNA copy number/cell content (Figure 2B), assessed by confocal microscopy and qPCR, respectively. Moreover, 3AB-OS displayed more fused elongated mitochondrial networks (Figure 2A, enlarged details), as reported for other mesenchymal cells relying on a glycolysis-dependent energy metabolism [23], whereas MG-63 cells showed mostly fragmented mitochondria, in agreement with morphology analysis reported by Palorini et al. [20]. Interestingly, evaluation of the basic reactive oxidant species (ROS) by the fluorescent probe DCF highlighted higher steady-state levels of ROS in CSCs with respect to parental cells (Figure 2C), likely contributing to the observed upregulation of glycolysis in 3AB-OS [24].

### 3.2. Effects of Energy Substrate Availability on Tumor Cell Proliferation

**Figure 3:**
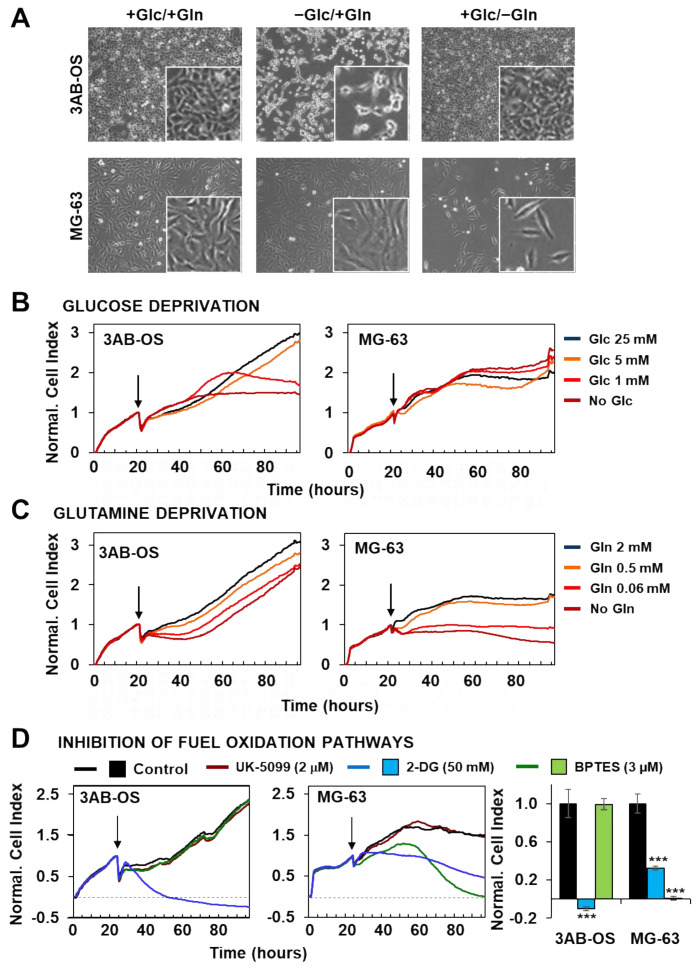
Effects of nutrient availability and metabolic pathway inhibition on osteosarcoma cell proliferation. (**A**) Phase-contrast images of 3AB-OS (upper panel) and MG-63 (lower panel) cultured for 48 h in three different conditions (from the left to the right): +Glc/+Gln, complete DMEM medium containing 25 mM glucose (Glc) and 2 mM glutamine (Gln); +Glc/−Gln, DMEM medium with 25 mM Glc, without Gln; −Glc/+Gln, DMEM medium with 2 mM Gln, without Glc. (**B**,**C**) Representative normalized cell index kinetics of 3AB-OS and MG-63 cells exposed to stepwise decrease in Glc or Gln concentration. Cells were grown in complete DMEM medium for approximately 24 h after seeding. Then medium was changed, and cells were treated for 72 h with DMEM medium containing different concentrations of Glc (**B**) or Gln (**C**) as indicated by the arrows. Each cell index value was normalized just before this starting point. (**D**) Normalized Cell Index traces of osteosarcoma cell lines incubated with either the glycolytic inhibitor 2-DG (50 mM) or the GLS1 inhibitor BPTES (2 µM) or the mitochondrial pyruvate carrier UK-5099 (2 μM) for 72 h. Cells were grown in complete DMEM medium and the day after treated with antimetabolites. The histogram (last graph on the right) displays different sensitivity to 2-DG or BPTES of 3AB-OS and MG-63 cells. NCI values have been normalized to the relevant control. Data are presented as mean ± SD; n = 3. Statistical significances are referred to control. *** *p* < 0.0001. Abbreviations: *2-DG*, 2-deoxy-D-glucose; *BPTES*, N,N′-[thiobis (2,1-ethanediyl-1,3,4-thiadiazole-5,2-diyl)] *bis*-benzeneacetamide.

Our previous findings suggested 3AB-OS and MG-63 cell lines relying primarily on glycolysis and oxidative phosphorylation, respectively, for energy production. Cell proliferation and energy producing metabolic pathways are strikingly linked cellular processes, as sharing common regulatory mechanisms [25]. In this frame, glutamine and glucose are essential nutrients and energy sources for cancer cell growth and survival and the impact of their removal from the culturing medium was tested. Figure 3A shows that removal of glucose affected specifically the growth of 3AB-OS CSC whereas removal of glutamine impacted specifically on that of MG-63 cells.

To further deepen the effects of selective energy substrate withdrawal on 3AB-OS and MG-63 proliferation we probed real time growth by xCELLigence System Cell Analyzer (RTCA), within a 72-h time window. 3AB-OS and MG-63 response to glucose reduction was evaluated in four different conditions: high glucose (25 mM); normal glucose (5 mM); low glucose (1 mM); no glucose (glutamine was supplied at 2 mM final concentration in the nutrient medium). 3AB-OS cell growth was dramatically delayed upon glucose deprivation, in a dose-dependent manner; on the other hand, glucose shortage did not exert any significant inhibitory effect on MG63 cell proliferation (Figure 3B).

Next the cell index kinetics was monitored in response to glutamine depletion, probing stepwise decrease of glutamine concentration (2, 0.5, 0.06 mM and no glutamine) in DMEM medium containing 25 mM glucose. If CSCs unveiled a delay followed by full recovery of the growth, differentiated osteosarcoma cells displayed great sensitivity to stepwise glutamine deprivation, exhibiting a progressive drop of the cell index (Figure 3C).

**Figure 4:**
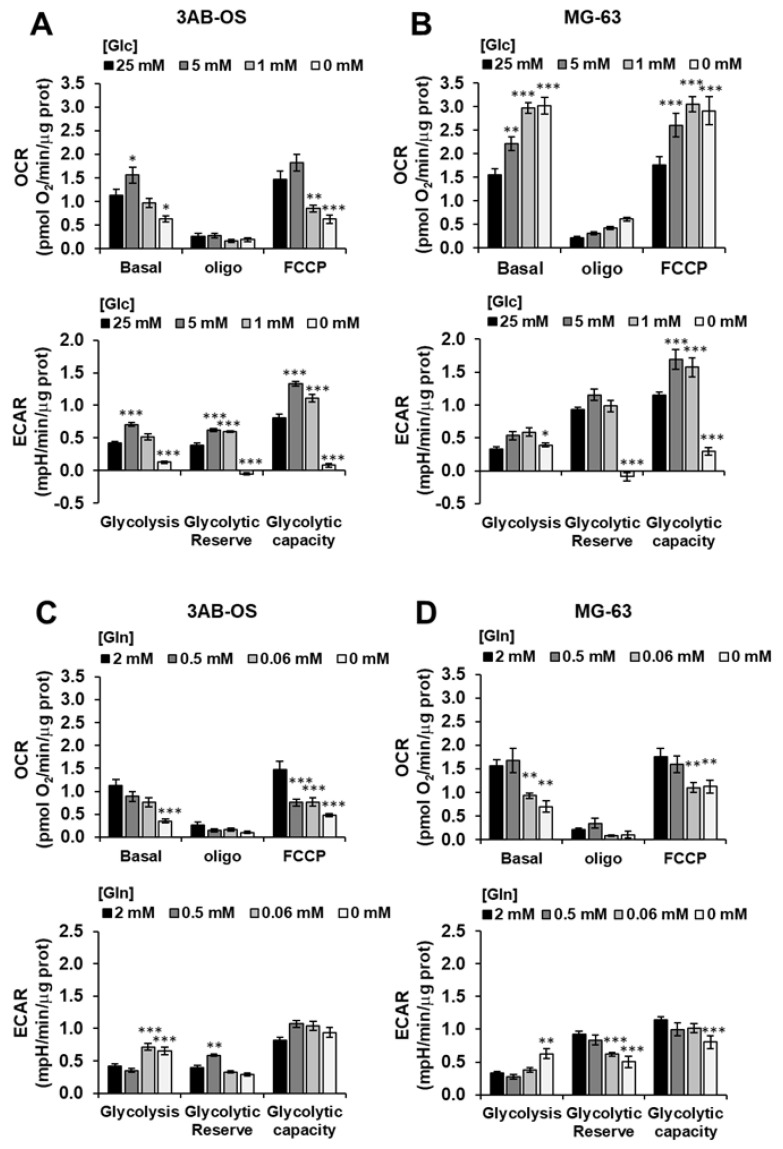
Effects of nutrient availability on the metabolic fluxes of osteosarcoma cell proliferation. 3AB-OS (**A**,**C**) and MG-63 (**B**,**D**) were incubated 48 h with the indicated different concentrations of glucose (Glc) or glutamine (Gln) and OCR and ECAR measured by Seahorse as in Figure 1A,B. Bars indicate mean ± SEM of 3 biological replicates each carried out in triplicate. Statistical significances are referred to control; * *p* < 0.05, ** *p* < 0.005, *** *p* < 0.001. Abbreviations: *OCR*, oxygen consumption rate; *ECAR*, extracellular acidification rate.

The above reported results were complemented with analysis of the metabolic fluxes under similar conditions of step wise energy substrate withdrawal after 48 h (Figure 4). It can be noted that glucose (Glc) deprivation caused in 3AB-OS CSC upregulation of both OCR and ECAR at 5 mM Glc that however reversed at more limited conditions of Glc regimen (Figure 4A). Conversely, in MG-63 cells stepwise starvation of Glc caused a substantial progressive increase of the OCR accompanied with enhanced ECAR at 5 and 1 mM Glc (Figure 4B). The result on the OCR in MG-63 refers to the inverted Crabtree effect, namely the capability of cells to optimize mitochondrial substrates oxidation when glucose availability becomes limiting [26,27,28]. Notably, the observed metabolic fitness of MG-63 is apparently absent in 3AB-OS CSCs. When the OS cell lines were challenged with glutamine withdrawal, 3AB-OS compensated a decreased in OCR enhancing ECAR (particularly at 0.06 mM Glc) whereas MG-63 did not (Figure 4C,D).

To further validate the reliance of the two cell lines on distinct metabolic pathways, we evaluated the effect of specific pharmacological inhibitors (Figure 3D). Indeed, cell viability was assessed by using the RTCA platform to evaluate cell growth rate in presence of (a) UK5099, which impairs glucose oxidation through blockage of the mitochondrial pyruvate carrier (MPC), (b) the glycolytic inhibitor 2-deoxy-D-glucose (2-DG), (c) BPTES (N,N′-[thiobis (2,1-ethanediyl-1,3,4-thiadiazole-5,2-diyl)] *bis*-benzeneacetamide), which is an allosteric inhibitor of glutaminase (GLS1), thereby interfering with the glutamine oxidation path. MPC inhibition produced no effect on cell proliferation in both cell lines. After exposure to 50 mM of 2-DG, 3AB-OS cells showed an immediate and rapid drop of cell index value below to 0, corresponding to cell death, while MG63 cells were less sensitive to 2-DG treatment. Finally, treatment with 3 µM BPTES induced proliferation arrest and cell death exclusively in differentiated osteosarcoma cells.

Taken together, these observations highlighted two distinct metabolic profiles for CSCs and differentiated cells. 3AB-OS CSCs are strikingly dependent on glycolysis for their proliferation and survival, as revealed by the cytotoxic effects exerted by glucose withdrawal or 2-DG-mediated glycolysis inhibition; lack of response to MPC blockage is a further evidence for glycolysis addiction and mitochondrial hypofunction in 3AB-OS. In agreement with the greater flexibility in glucose usage, MG63 cell growth and survival is linked to glutamine availability, as proven by cell growth arrest due to either glutamine deprivation or inhibition of glutamine oxidation.

### 3.3. Cell Viability Effects of Cisplatin Combined with Energy Substrate Deprivation

In the light of our previous findings, we explored potential additive antiproliferative effects of cisplatin with fuel substrate deprivation on osteosarcoma cell lines. Combination therapy involving chemotherapy and metabolic targeting may (a) result in improved cell responsiveness and remarkable antitumor effect, and (b) allow for reduction of the effective drug concentration, thereby limiting side effects and toxicity.

**Figure 5:**
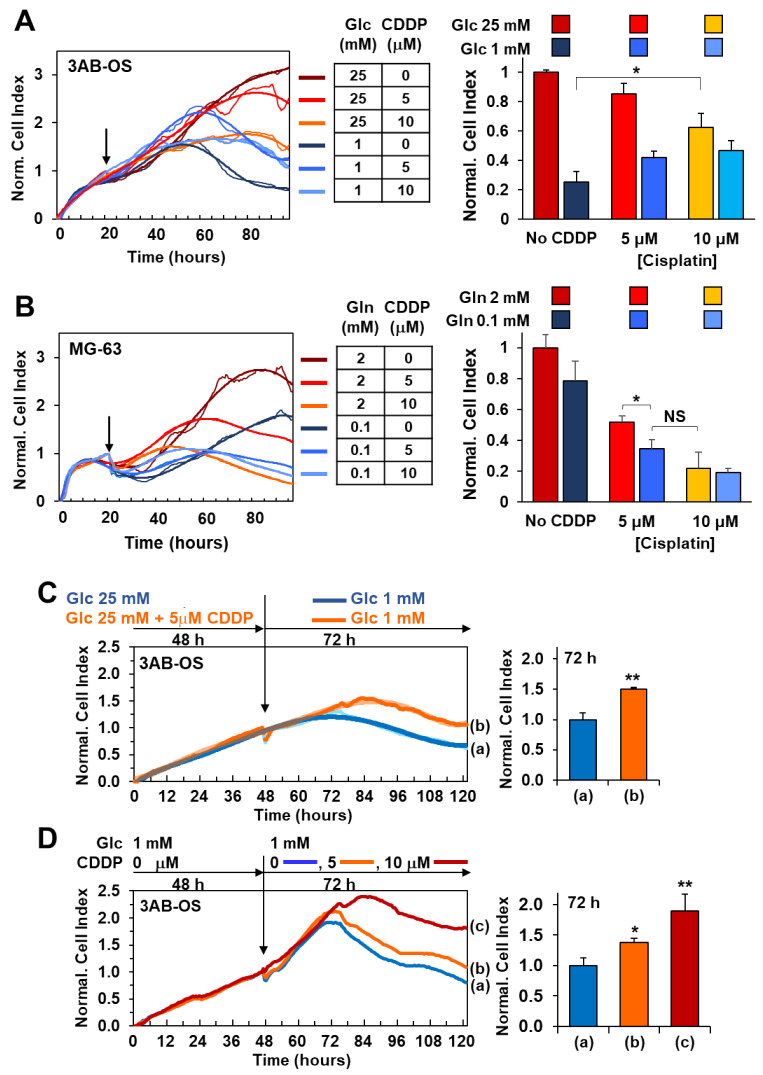
Cell viability effects of cisplatin combined with energy substrate deprivation. (**A**) Real-time monitoring of 3AB-OS cell growth after 72 h treatment with cisplatin in high (25 mM)/low (1 mM) glucose conditions. Cells were cultured under normal growth condition (DMEM medium with 25 mM Glc and 2 mM Gln) for approximately 24 h, and, after medium change, exposed to 5 and 10 µM cisplatin (CDDP) in complete DMEM medium supplemented with either 25 mM or 1 mM Glc. Panel on the left: representative NCI traces. Bar histogram on the right: NCI values normalized to controls represented by untreated cells (No CDDP), cultured under normal growth condition. Data are presented as mean ± SD of three independent experiments (biological replicates). * *p* < 0.05. (**B**) Real-time monitoring of MG-63 cells after 72 h treatment with CDDP in high (2 mM)/low (0.1 mM) glutamine conditions. Cells were cultured under normal growth condition (DMEM medium with 25 mM Glc and 2 mM Gln) for approximately 24 h, and, after medium change, exposed to 5 and 10 µM cisplatin in complete DMEM medium supplemented with either 2 mM or 0.1 mM Gln. Panel on the left: representative NCI traces. (**B**) Bar histogram: NCI values normalized to controls represented by untreated cells (No CDDP), cultured under normal growth condition. Data are presented as mean ± SD of three independent experiments (biological replicates). * *p* < 0.05. (**C**,**D**), antagonist effects of glucose shortage and cisplatin administration on 3AB-OS proliferation. (**C**) Response of CDDP-pretreated 3AB-OS cells to glucose shortage. Left panel, representative growth curves of 3AB-OS cells pretreated for 2 days in high glucose +/− 5 µM CDDP and then incubated in low glucose (without CDDP) for 72 h. Right panel, bar histogram: NCI values of CDDP-pretreated 3AB-OS cells relative to control (cells not pretreated with CDDP) after 72 h treatment. (**D**) Sensitivity of low-glucose pretreated 3AB-OS cells to CDDP. Left panel, representative growth curves of 3AB-OS cells pretreated with low glucose for 2 days and then exposed to CDDP (5 and 10 µM) or vehicle in low glucose nutrient medium for 72 h. Right panel, bar histogram: NCI values of low glucose-pretreated 3AB-OS cells relative to control (Glc 1 mM) after 72 h treatment. All data are presented as mean ± SD of three independent experiments. * *p* < 0.05; ** *p* < 0.01. Statistical significances are referred to the controls.

Cell viability was real-time monitored within a 72-h time window after exposure to (a) cisplatin in both OS cell lines, (b) glucose deprivation and (c) combination of cisplatin with glucose deprivation in 3AB-OS cells, (d) glutamine deprivation and (e) combination of cisplatin with glutamine deprivation in MG63 cells (Figure 5A,B). MG-63 cells resulted to be more sensitive than 3AB-OS to 5 and 10 µM cisplatin treatments, thus giving a further evidence for the existence of more effective chemoresistance mechanisms in CSCs than differentiated cells.

MG-63 cell proliferation was slightly delayed after lowering glutamine concentration from 2 mM (commonly used for in vitro culture) to 0.1 mM, although being about 6-fold lower than the physiological range (0.6 mM). Co-treatment with 5 and 10 µM cisplatin at low glutamine regimen resulted in potentiation of the MG-63 cells growth decrease as compared to 0.1 mM Gln treatment alone which did not elicit any significant response (Figure 5B). To note treatment with 5 µM cisplatin at low Gln concentration caused an antitumoral effect comparable to that of the higher-dose treatment of cisplatin (10 µM) at high Gln concentration.

**Figure 6:**
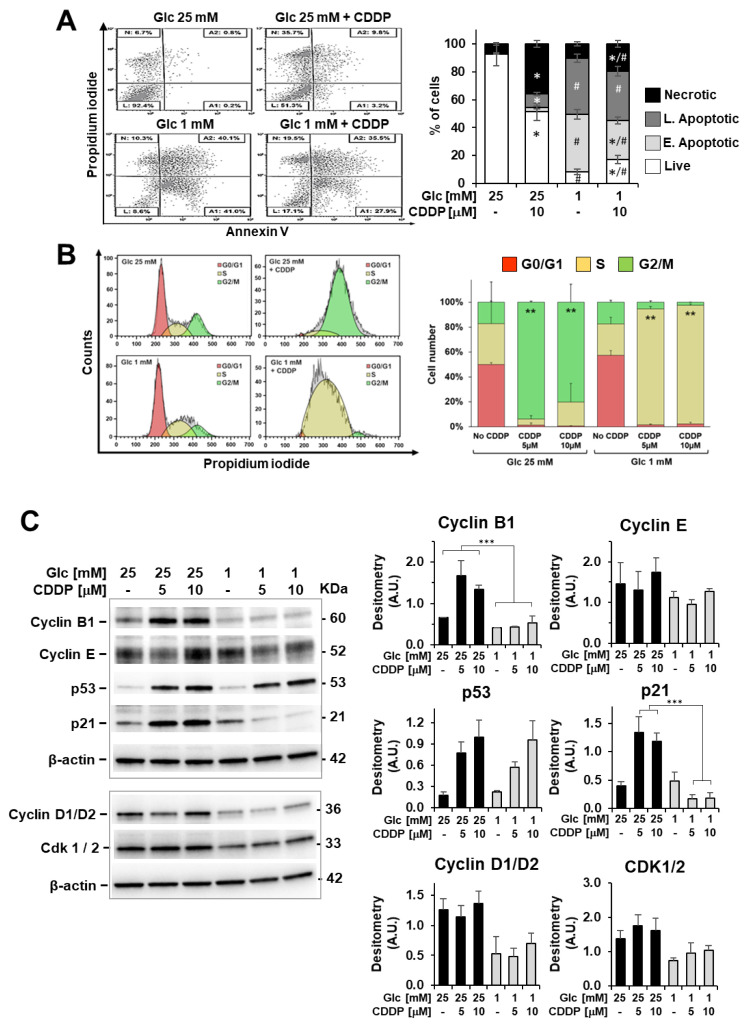
Cisplatin (CDDP) effects on apoptosis induction and cell-cycle distribution in 3AB-OS cells under high and low glucose growth conditions. (**A**) Flow cytometric detection of apoptosis and necrosis with Annexin-V-fluorescein isothiocyanate (FITC) and propidium iodide staining in 3AB-OS cells after 72 h exposure to 10 µM CDDP in high (25 mM) and low (1 mM) glucose. Dot plots on the left show a single representative experiment; histogram on the right shows the statistical analysis of three independent biological replicates. Abbreviations: *L*, live cells; *A1*, early apoptotic cells; *A2*, late apoptotic cells; *N*, necrotic cells. *, *p* < 0.05 vs. cells grown at high and low Glc but in the absence of CDDP; #, *p* < 0.05 vs. cells grown at high and low Glc but in the presence of CDDP. (**B**) Cell cycle analysis through PI staining and flow cytometry of 3AB-OS cells after 48 h treatment with 10 µM CDDP in 25 mM and 1 mM glucose media. Cell cycle histogram plots show a single representative experiment (left panel). Changes in cell cycle distribution of 3AB-OS cells exposed to CDDP (5 and 10 µM) or vehicle in high and low glucose conditions for 48 h (right panel). Data are presented as mean ± SD; n = 3. Statistical significances are referred to the relevant controls. ** *p* < 0.01. (**C**) Protein expression of cell cycle-controlling factors. A representative cropped Western blotting of total 3AB-OS cells extract is shown; cells were treated at high and low Glc with CDDP as indicated for 48 h. The histograms show statistical evaluation of the densitometric analysis (normalized to β-actin) for the indicated proteins; bar values are means ± SEM of three independent experiments. *** *p* < 0.0005.

Cell viability assays unveiled that lowering glucose concentration from 25 mM (high glucose) to 1 mM (low glucose) is more effective than 10 µM cisplatin administration in high glucose to inhibit 3AB-OS proliferation (Figure 5A). Moreover, cisplatin-induced cell death is mainly necrotic rather than apoptotic, whereas glucose shortage acts through a pro-apoptotic mechanism, as shown in annexin V-FITC/PI assays (Figure 6A). Remarkably, 5 and 10 µM cisplatin treatments did not act synergistically either with 2.5 mM (not shown) or 1 mM glucose conditions in inhibiting proliferation of 3AB-OS CSCs. Unexpectedly, cisplatin exposure prolonged 3AB-OS cell survival in 1 mM glucose DMEM medium as compared to 1 mM glucose treatment alone. Indeed, 3AB-OS cell index increased after 72 h treatment with 5 or 10 µM cisplatin in 1 mM glucose DMEM medium (Figure 5A). Even if cisplatin-induced prosurvival effects were more evident in 1 mM glucose medium, CSCs exhibited a similar response in 2.5 mM glucose in the presence of cisplatin (not shown).

### 3.4. Cisplatin Attenuates Sensitivity to Low Glucose as Well as Glucose Shortage Elicits Cisplatin Resistance

To evaluate lack of cytotoxicity of cisplatin in low glucose, we assessed relative amounts of necrotic, apoptotic, and live cells by the annexin V-FITC/PI assay. After 72 h incubation with 10 µM cisplatin in 1mM glucose medium, the percentage of live stem cells was higher (2X) than that of cells treated exclusively with 1 mM glucose, with a concomitant decrease of apoptotic cell death (Figure 6A).

These observations suggest an antagonist effects of the combined use of glucose withdrawal and chemotherapy by cisplatin, indicating that hampering glucose metabolism is not a good strategy for sensitizing 3AB-OS osteosarcoma stem cells to cisplatin. Moreover, these results raise the question of whether glucose deprivation triggers resistance mechanisms to cisplatin or, on the other hand, cisplatin weakens cell responsiveness to glucose withdrawal within 3AB-OS CSCs.

In the attempt to trace back this antagonist effect to cisplatin- and/or low glucose-induced molecular mechanisms, 3AB-OS CSCs were pre-treated for 48 h with 5 µM cisplatin in high glucose and, then, after medium removal, exposed to 1 mM glucose to evaluate cell sensitivity to glucose deprivation by real-time monitoring of cell growth for 72 h (Figure 5C). This concentration of cisplatin (5 µM) was chosen for pretreatment as it exhibited a negligible effect on cell growth. Cell index kinetics of cisplatin-pretreated cells revealed a delayed drop in the proliferation-curve slope compared to non-pretreated cells, thus accounting for a cisplatin-induced decrease in sensitivity to glucose withdrawal (Figure 5C).

In parallel, in order to check if, in turn, glucose deprivation triggered cisplatin chemoresistance, 3AB-OS CSCs were grown under low glucose condition (1 mM) for 48 h and, then, exposed to either 5 or 10 µM cisplatin in low glucose nutrient medium for 72 h. Real time monitoring of cell proliferation unveiled that low glucose pre-treated 3AB-OS cells resulted to be immune to drug-treatment; moreover, cisplatin addition ameliorates cell survival in low glucose medium, in a dose dependent manner (Figure 5D). After 72 h treatment with 10 µM cisplatin in low glucose, CSCs normalized cell index was twice the control value. Interestingly, lack of cisplatin cytotoxicity was observed only when low glucose pre-treated CSCs were simultaneously exposed to cisplatin and low glucose during treatment. Indeed, CSCs sensitivity to cisplatin was restored when low-glucose pre-treated CSCs were incubated with cisplatin in high glucose medium (data not shown).

Aiming to understand acquired cisplatin-resistance of CSCs in 1 mM glucose, we compared cisplatin effects on apoptosis induction, cell-cycle distribution, and ROS production in high and low glucose nutrient media. In high glucose, cisplatin exposure led to necrotic cell death and cell accumulation in G2/M phase after 48 h (Figure 6A,B). In low glucose, cisplatin-induced necrosis was almost negligible, and a G2/M cell cycle arrest was lacking, with CSCs experiencing a permanent S-phase status after 24, 48 and 72 h treatment (Figure 6A,B). Accordingly, cyclin B1 accumulation in stem cells was detected after 48 h exposure to CDDP only in high glucose (Figure 6C), and this is indicative of defective cell-cycle progression from G2 through M phase and drug-induced mitotic arrest [29].

Following DNA damage caused by chemotherapy or genotoxic insults, the p53-p21 axis orchestrates mobilization of the DNA damage response together with modulation of cell cycle checkpoints to allow DNA repair and promote maintenance of genome stability [30]. To explain the different cisplatin effects on cell cycle progression in high and low glucose culture media, we examined the p53 and p21 expression levels after 48-h treatment by immunoblotting. Cisplatin-treated 3AB-OS cells exhibited higher p53 protein levels as compared to relevant controls, both in high and low glucose, as expected (Figure 6C). However, if homogenous p53 responses could be noticed regardless of glucose concentration, cisplatin treatment resulted in opposite p21 dynamics in high- and low-glucose conditions, although p21 is a well-known transcriptional target of p53. In high glucose, p53-dependent expression of p21 mediates the observed G2/M cell cycle arrest after cisplatin treatment, as widely reported in the literature [30]. On the other hand, in low glucose, the amount of p21 was reduced in cisplatin-treated cells, which is consistent with a S-phase arrest [31]. Notably, the absence of G2/M cell cycle arrest has been already associated to cisplatin resistance in non-small cell lung cancer (NSCLC) cell line A549rCDDP2000 [32] as well as S-phase blockage exerts cytoprotective effects by allowing repair mechanisms to re-establish DNA integrity [31,33].

**Figure 7:**
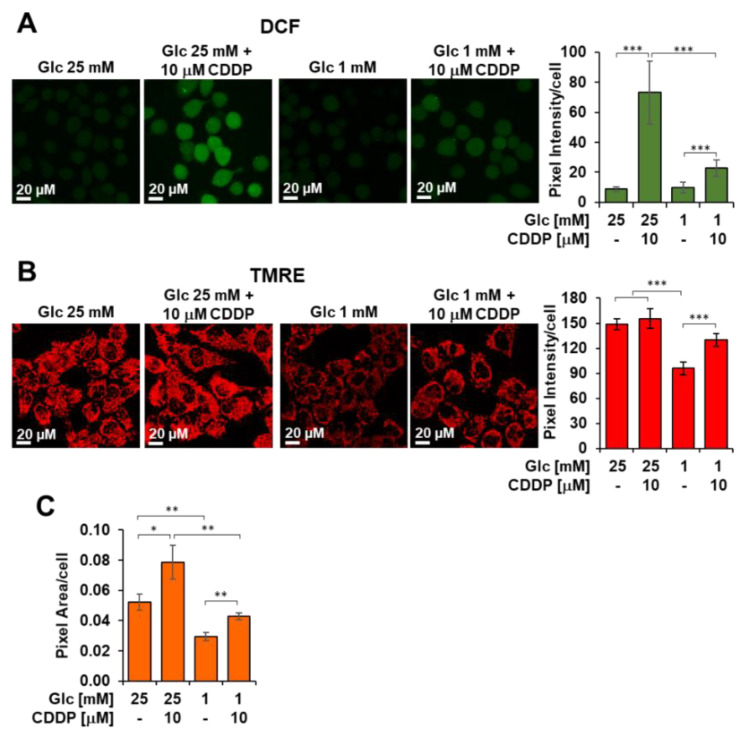
Cisplatin (CDDP) effects on ROS production and mitochondrial membrane potential in 3AB-OS under high and low glucose growth conditions. Cells were treated with the CDDP or vehicle at low and high glucose (Glc) as indicated and analysed after 48 h. (**A**) Laser scanning confocal microscopy (LSCM) of ROS production by the fluorescent probe dichlorofluorescein (DCF). Left panel shows representative LSCM images. Bar histogram on the right shows statistical analysis of the DCF pixel intensity/cell; values are means ±  SD of three independent biological replicates; *** *p*  <  0.001. Ten randomly chosen optical fields/sample each containing about 15 cells were analyzed by ImageJ. (**B**) LSCM analysis of mitochondrial transmembrane potential (ΔΨ_m_) by the fluorescent probe TMRE (tetramethylrhodamine, ethyl ester). Left panel shows representative LSCM images. Bar histogram on the right shows statistical analysis of the TMRE pixel intensity/cell; values are means ±  SD of three independent biological replicates; *** *p*  <  0.001. Ten randomly chosen optical fields/sample each containing about 15 cells were analyzed by ImageJ. (**C**) Bar histogram showing the overall TMRE-related pixel area/cell; values are means ±  SD of three independent biological replicates; * *p*  <  0.05, ** *p*  <  0.01.

Next, we evaluated if cisplatin was able to rise cellular ROS levels at mitochondrial level by confocal microscopy imaging using the peroxide probe DCF (Figure 7A). Together with the formation of cross-links in DNA, ROS generation is acknowledged to underlie cisplatin-induced cell injury. After incubation with cisplatin, 3AB-OS displayed a larger increase of the DCF signal in 25 mM glucose as compared to cells treated with cisplatin in 1 mM glucose, where ROS levels appeared to be similar to those from untreated cells. These findings suggested that low glucose hampered cisplatin-signaling cascade bridged to cytotoxicity to take place within CSCs. Particularly, the observation that low glucose inhibits the mitochondrial-dependent ROS response to cisplatin, indicated 3AB-OS CSCs could elude the cytotoxic potential of cisplatin by reducing its accumulation in mitochondria. In the cytosol, cisplatin reacts spontaneously with water molecules to generate membrane-permeable cationic adducts, which are promptly accumulated inside mitochondria, owing to their negative inner membrane potential [34].

By using confocal microscopy imaging with the TMRE probe, we could observe a significant reduction of the inner membrane potential in CSCs seeded in low glucose as compared to those in high glucose (Figure 7B). This alteration of mitochondrial potential is likely to reduce cisplatin uptake and prevent activation of mitochondrial ROS signaling.

**Figure 8:**
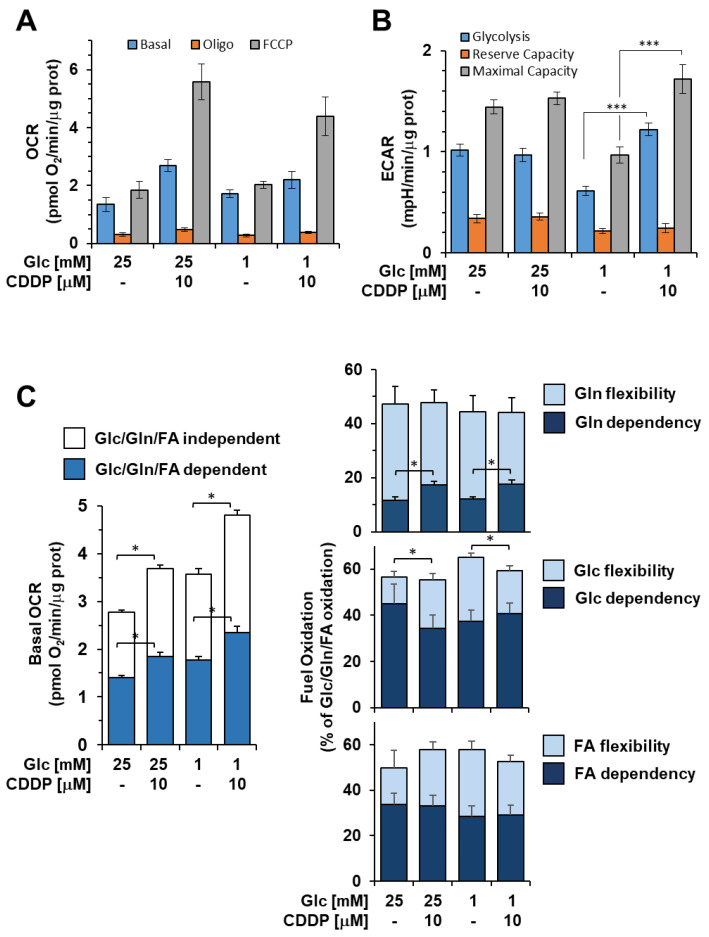
Cisplatin (CDDP) effects on metabolic fluxes and mitochondrial respiratory activities 3AB-OS CSCs. Cells were incubated 48 h with 10 μM CDDP under high and low glucose growth conditions as indicated and subjected to Seahorse analysis for OCR (**A**) and ECAR (**B**) as detailed in Figure 1A,B. Bar values are means ± SEM of three independent biological replicates each carried out in triplicate; ***, *p* < 0.001. (**C**) Mitochondrial dependency on and flexibility for oxidation of glucose (Glc), glutamine (Gln), and long chain fatty acids (FA) in presence or absence of CDDP, in high and low glucose nutrient media. The bar histogram on the left shows the combined contribution of glucose (Glc), glutamine (Gln) and long chain fatty acids (FA) to the basal OCR; the residual activity is defined as Glc/Gln/FA-independent OCR. The tripartite bar histograms on the right show the dependency and flexibility of the 3AB-OS OCR relatively to one or the other of Glc, Gln and FA as respiratory substrates. Bar values are means ± SEM of three independent biological replicates each carried out in triplicate; *, *p* < 0.005. See Materials and Methods for further details. Abbreviations: *oligo*, oligomycin; *FCCP*, carbonyl cyanide 4-(trifluoromethoxy)phenylhydrazone; *OCR*, oxygen consumption rate; *ECAR*, extracellular acidification rate.

If the TMRE stained compartment per cell is considered as a measure of the cellular mass density, it can be noted that 48 h growth of 3AB-OS CSC at low-Glc (i.e., 1 mM) caused a significant 45% decrease of it (Figure 7C). Interestingly, treatment with 10 µM cisplatin elicited an increase of the mitochondria-related fluorescent signal irrespective of the Glc regimen. These results correlated with the observed increase of the maximal OCR, that is a measure of the respiratory chain content, following cisplatin treatment under both high- and low-Glc growth conditions (Figure 8A see ahead).

### 3.5. Cisplatin Enhances Glycolysis, Mitochondrial OxPhos and Metabolic Flexibility in 3AB-OS CSCs

Our results led to the hypothesis of a cisplatin-driven metabolic reprograming towards a more oxidative metabolism in osteosarcoma stem cells, which were previously shown to have a remarkable glycolytic phenotype. Therefore, we proceeded to outline metabolic changes associated with enhanced resistance to glucose depletion and prolonged cell survival, upon cisplatin exposure. 3AB-OS CSCs were pre-treated for 48 h with 5 µM cisplatin in high/low glucose conditions and then, evaluated for bioenergetic competence/efficiency of glycolysis and mitochondrial respiration, using the Seahorse extracellular flux analyzer. While the rate of glycolysis, calculated as ECAR, was substantially unaffected in high glucose (Figure 8B), cisplatin exposure elicited a mechanism for cells to promote glycolysis and cope with the energy crisis under glucose limiting conditions.

In parallel, high resolution oximetry revealed that cisplatin caused a significant higher basal OCR in 3AB-OS CSCs, regardless of glucose availability (Figure 8A,C). Particularly, cisplatin was shown to increase OCR dependency from glutamine oxidation, while glucose- and fatty acid-dependency remained unchanged (Figure 8C). More importantly, cisplatin enhanced metabolic flexibility in substrate usage to sustain mitochondrial respiration (Figure 8C). Therefore, after cisplatin incubation, CSCs mitochondria developed a greater ability to compensate for the inhibition of glucose, fatty acid, and glutamine oxidation by using alternative carbon sources, thereby improving adaptability to environmental changes. In addition, it is worthy to note that CSCs exhibited the highest basal OCR as well as the greatest metabolic flexibility after exposure to combined use of chemotherapy by cisplatin and glucose shortage (Figure 8C). Cisplatin-induced increase in mitochondrial function is consistent with the lower glycolytic flexibility exhibited by 3AB-OS CSCs during glucose starvation.

Overall, the enhanced glycolytic flux as well as the improvement of mitochondrial OxPhos and plasticity can provide additional energy to sustain cell proliferation for a longer time in low glucose conditions. Therefore, these metabolic changes may contribute to cisplatin-prosurvival effects observed in 1 mM glucose nutrient medium.

To explain the mechanism behind upregulation of mitochondrial OxPhos, we studied expression levels of sirtuin 1 (Sirt1) and sirtuin 3 (Sirt3), which are known to exert a dual control on mitochondrial biogenesis and activity [35]. Sirt1 is mainly localized in the nucleus and triggers the peroxisome proliferator activated receptor γ co-activator 1α (PGC-1α)-mediated transcription of nuclear and mitochondrial genes encoding for proteins fostering mitochondrial biogenesis and OxPhos. Sirt3 is mainly located in the mitochondrial compartment, where it directly drives synthesis of proteins involved in OXPHOS, tricarboxylic acid (TCA) cycle and fatty-acid oxidation [35].

**Figure 9:**
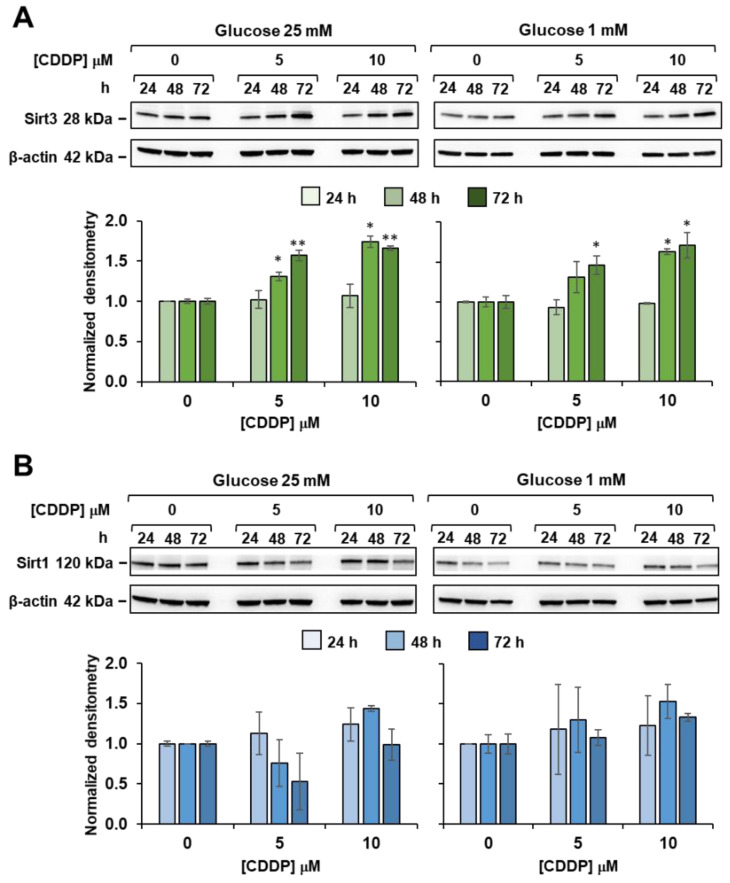
Effect of cisplatin on sirtuins expression in 3AB-OS under high and low glucose growth conditions. Cells were treated with different concentrations of cisplatin (CDDP) and glucose regimen as indicated; whole cell proteins were extracted at the specified time points and subjected to SDS-PAGE and blotted toward either SIRT3 (**A**) or SIRT1 (**B**). Upper panels in (**A**,**B**) are representative cropped immunoblots of Sirt3 and Sirt1 respectively; lower panels are graph bars showing the average ± SD of data resulting from densitometric analysis (normalized to β-actin) of three independent biological replicates under each condition. Statistical significances are referred to the relevant controls; * *p* < 0.05, ** *p* < 0.01.

Western blot analyses (Figure 9A) showed that 5 and 10 µM cisplatin treatments elicited Sirt3 upregulation at 48 and 72 h, in both high and low glucose media. On the other hand, no significant differences were detected in the amounts of Sirt1 from cisplatin-treated 3AB-OS (Figure 9B). A significant increase in Sirt1 expression could be noticed only after 48 h exposure to 10 µM cisplatin in high glucose. However, as mitochondrial OCR remained unchanged after exposure to cisplatin, in the presence or absence of the Sirt1 inhibitor EX-527 (data not shown), it could be argued that the observed metabolic reprogramming was a Sirt1-independent process.

In the light of these findings, it can be speculated that cisplatin triggers Sirt3-mediated effects even at the lowest dose (5 µM) to induce higher rate of OxPhos in 3AB-OS CSCs. Indeed, it is known that Sirt3 is a stress-responsive deacetylase and may be upregulated upon DNA damage induced by cisplatin, in order to activate DNA repair mechanisms and protect from cell death [36,37].

### 3.6. Glucose Deprivation Induces Activation of Autophagy to Sustain Cell Proliferation

Insufficient supply of nutrients often leads to activation of survival strategies within cancer cells. Following the failure of the combined use of cisplatin and glucose shortage, we investigated alternative targets in 3AB-OS CSCs in order to enhance their metabolic vulnerability during starvation.

**Figure 10:**
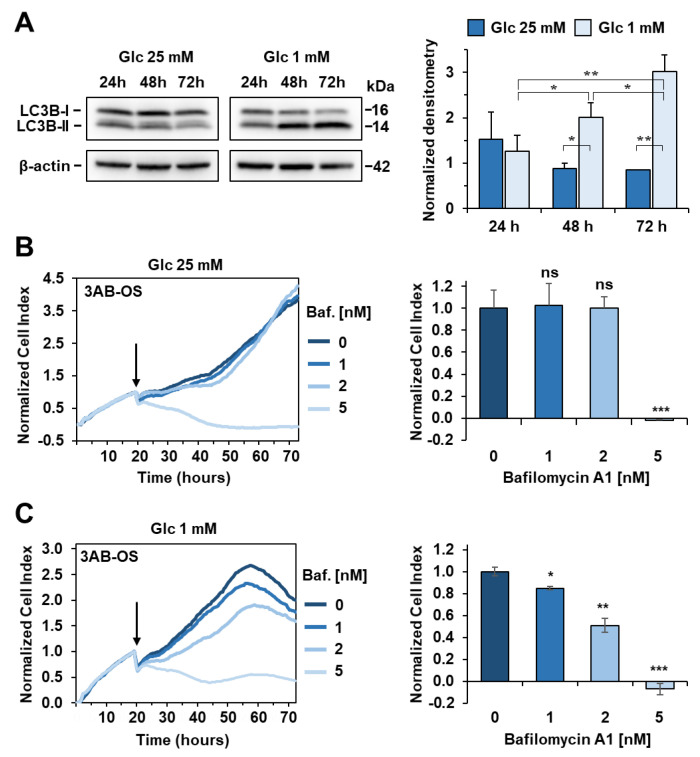
Activation of autophagy flux during glucose (Glc) starvation in 3AB-OS CSCs. (**A**) Left panel: representative immunoblotting for protein expression levels of LC3B in high and low glucose, after incubation at 24 h, 48 h and 72 h; β-actin was used as loading control. Right panel: densitometric analysis displaying LC3B-II/LC3B-I ratio (normalized to β-actin), expressed as mean ± SD of three independent experiments (* *p* < 0.05, ** *p* < 0.01). (**B**) Left panel: representative NCI traces of 3AB-OS CSCs after exposure to 1, 2, 5 nM bafilomycin A1 (Baf) and vehicle (control). CSCs were cultured for approximately 24 h in 25 mM Glc DMEM medium, and then treated with the indicated concentrations of Baf in high glucose for 48 h. Right panel: the bar histogram shows NCI variations after 24 h exposure to Baf or vehicle. Data are expressed as mean ± SD of three independent experiments. Statistical significances are referred to control; *** *p* < 0.0001. (**C**) Left panel: representative NCI traces of 3AB-OS CSCs after exposure to 1, 2, 5 nM bafilomycin A1 (Baf) and vehicle (control). CSCs were cultured for approximately 24 h in 1 mM Glc DMEM medium, and then treated with the indicated concentrations of Baf in low glucose for 48 h. Right panel: the bar histogram shows NCI variations after 24 h exposure to Baf or vehicle. Data are expressed as mean ± SD of three independent experiments. Statistical significances are referred to control; * *p* < 0.05, ** *p* < 0.01, *** *p* < 0.0001.

In this scenario, we speculated that glucose withdrawal might enhance autophagy, which in turn could maintain energy homeostasis, by recycling proteins and organelles to provide metabolic substrates. Thus, we analysed autophagy under high and low glucose conditions. Twenty-four hours after seeding in 25 mM glucose DMEM medium, cells were switched to either high or low glucose conditions to monitor autophagy after 24, 48 and 72 h by western blot analyses (Figure 10A). Consistent with our hypothesis, glucose withdrawal resulted in induced conversion of LC3B, a key autophagy marker, at both 48 h and 72 h. Then, we investigated whether autophagy interference could negatively affect cell proliferation in 1 mM glucose. Toward this aim, we subjected CSCs to 1, 2 nM and 5 nM bafilomycin A1, which is a selective inhibitor of the late phase of autophagy (Figure 10B). 3AB-OS cells were treated with bafilomycin A1 almost after 24 h incubation in 1 mM glucose, when autophagy appeared to be induced according to WB analyses. CSCs experienced slightly, but significantly delayed cell growth in presence of 2 nM bafilomycin A1. Treatment with 5 nM bafilomycin was shown to be excessively cytotoxic, thereby suggesting additional mechanisms other than autophagy inhibition could be responsible for such a rapid cell death at the highest dose tested.

## 4. Discussion

Malignant tumors display heterogeneous populations of cells, such as stem and differentiated cells, experiencing various states of differentiation and proliferation as well as different metabolic addictions. Therefore, even if being effective against differentiated and high proliferative cancer cells, conventional treatments need to be implemented to target CSCs for complete tumor eradication. In this frame, we focused on comparative metabolic profiling of two osteosarcoma-derived cell lines, the 3AB-OS cancer stem cells and the parental MG-63 cells, featuring two distinct metabolic phenotypes. Selective substrate deprivation or tailored manipulation of energy pathways clearly show reliance of MG-63 on glutamine oxidation and of 3AB-OS on glycolysis. Based upon these findings, we have demonstrated that (a) fine-tuning of glutamine represents a complementary treatment to sensitize MG-63 differentiated cells to chemotherapy by cisplatin whereas (b) cisplatin administration under selective withdrawal of glucose results into a paradoxical pro-survival and antioxidant outcome, leading to CSCs maintenance. Our study indicate that the efficacy of specific metabolite starvation combined with chemotherapeutic drugs depends on the cancer compartment and suggests caution in using it as a generalizable curative strategy.

### 4.1. Metabolic Heterogeneity of 3AB-OS and MG63 Osteosarcoma Cells Requires Distinct Therapeutic Approaches

3AB-OS cancer cells represent a distinct mesenchymal stem subpopulation isolated from the human osteosarcoma MG-63 cell line. CSCs feature a distinct metabolic phenotype from their differentiated counterpart. Indeed, 3AB-OS CSCs rely mostly on aerobic glycolysis for ATP synthesis, proliferation and survival, as highlighted by increased ECAR and higher sensitivity to glucose withdrawal or 2-DG-mediated glycolysis inhibition, with respect to MG-63 cells. Differently from parental cells, 3AB-OS cells display lower OCR and partially sustain a mitochondrial function with higher dependency from glucose oxidation, under normal growth conditions. Even if the fate of glucose intake is mainly fermentative in 3AB-OS cells, it could be partially directed towards the TCA cycle to provide building blocks for anabolic needs instead of energy supply. Indeed, CSCs growth resulted to be unaffected by blockage of the mitochondrial pyruvate carrier, thus providing a further evidence for glycolysis addiction and mitochondrial hypofunction in 3AB-OS. Although also in differentiated cancer cells inhibition of the mitochondrial pyruvate carrier does not appear to affect cell growth nevertheless substantial mitochondrial respiration is sustained by glutamine fueling. So far, our results confirm that bioenergetics of osteosarcoma stem cells is similar to that described for normal stem cells, where acceleration of glycolysis (i.e., the Warburg effect) and reduced rate of OxPhos has been shown to preserve the undifferentiated state and the self-renewal capacity [38,39]. Indeed, stem cells experiencing a metabolic switch towards OxPhos may undergo differentiation [40]. In this perspective, higher steady state levels of ROS detected in 3AB-OS rather than parental cells may enhance the metabolic flux through glycolysis and exert a negative feedback influence on mitochondrial activity, thereby minimizing further ROS generation and contributing to stemness maintenance.

Conversely, MG63 cells showed a greater reliance on mitochondrial respiration. Our results highlight glutamine to play a vital role for cellular growth of differentiated MG-63 cells, as glutamine withdrawal and inhibition of glutaminolysis dramatically impacted cell viability. Blocking glutamine oxidation impairs OxPhos, by reducing availability of intermediates of TCA cycle, and, at the same time, weakens ROS detoxification capability, through downregulation of GSH synthesis and NAPDH equivalents production [41].

Taken together, our observations shed light on the metabolic heterogeneity of the human osteosarcoma MG-63 cell line, consisting of (a) a large population of differentiated cells, with glucose-independent metabolism and glutamine-addiction, and (b) a small subset of highly glycolytic mesenchymal stem cells (i.e., 3AB-OS) whose survival is linked to glucose availability and insensitive to glutamine shortage. These differences cannot be overlooked in order to design a tailored-metabolic approach for a successful and complete tumor eradication.

### 4.2. Cisplatin Enhances 3AB-OS CSCs Metabolic Plasticity and Resistance to Glucose Starvation

In the light of the observed metabolic vulnerabilities of the two osteosarcoma cell lines, we assessed potential synergistic effects of energy substrate withdrawal and a first-line chemotherapeutic agent for treating osteosarcoma, i.e., cisplatin, aiming to provide the rationale for a combination therapy which integrates chemotherapy with tailored manipulation of energetic pathways. This approach resulted to be successful exclusively in parental MG-63 cells. Glutamine shortage and simultaneous exposure to 5 µM cisplatin turned out to produce a significantly potentiated antitumor effect, comparable to that of the single high-dose treatment with cisplatin (10 µM). These results indicate that hampering glutamine metabolism synergizes with cisplatin, thereby allowing for reduction of the effective drug concentration.

On the other hand, 3AB-OS CSCs exhibited a completely different behavior. They resulted to be more resistant to cisplatin treatment than MG-63 parental cells in normal growth conditions, confirming the role of CSCs in triggering chemoresistance. Notably, glucose shortage (and 2-DG-mediated inhibition of glycolysis) turned out to be more effective than 10 µM cisplatin treatment in slowing cellular growth rate in 3AB-OS cells.

However, cisplatin exposure was shown to prolong CSCs survival during glucose starvation as compared to low glucose treatment alone, thus highlighting the onset of antagonist effects of energy substrate withdrawal and cisplatin administration on 3AB-OS proliferation. Our results indicated that low glucose elicited resistance to 10 µM cisplatin, as confocal imaging depicted the absence of a mitochondrial-dependent ROS response, which is a relevant module of cisplatin-anticancer activity [42]. We hypothesized lack of cytotoxicity could be traced back to a reduced intramitochondrial accumulation of cisplatin. Indeed, glucose shortage was shown to decrease mitochondrial transmembrane potential (ΔΨ_m_), thereby hampering passive diffusion of cationic adducts of cisplatin across the mitochondrial membrane, as reported also by Qian et al. [34]. Moreover, alteration of 3AB-OS mitochondrial density, after prolonged exposure to 1 mM glucose, has been already described by Palorini et al. [20], and this may represent a cofactor in the development of chemoresistance [43]. As a consequence, low glucose prevents cisplatin from inducing both an extensive DNA damage and a ROS-mediated injury at a mitochondrial level. Overall, low glucose-mediated inhibition of the mitochondrial-dependent ROS response to cisplatin attenuates its ability to tilt redox homeostasis in CSCs, which is consistent with the lack of cellular necrosis and G2/M cell cycle arrest which, conversely, are related to cisplatin cytotoxicity observed in normal growth conditions (high glucose).

In low glucose, 3AB-OS cells exhibited obviously a slower growth rate, as indicated (a) by lower levels of cyclins D1/D2 and CDK1/2 (Figure 6C) and (b) increased cell doubling times in viability assays with respect to cells growing in high glucose. As a consequence, it can be argued that CSCs experience mild DNA damage accumulation after chemotherapy by cisplatin in low glucose nutrient media. Therefore, CSCs may show low p21 levels (or even its extensive degradation) to delay cell cycle progression in S phase and allow for repairing DNA lesions and restarting DNA synthesis [31,44].

On the contrary, in high glucose CSCs undergo extensive DNA damage upon chemotherapy as growing faster and sense these accumulating lesions through activation of the p53/p21 axis, which lead to mitotic arrest and cell death.

In low glucose, 3AB-OS CSCs are able to engage putative mechanisms transducing prosurvival signals in response to cisplatin, thereby prevailing over cisplatin-induced cellular damage. Cisplatin enhances glycolysis, mitochondrial OxPhos as well as CSCs plasticity during glucose starvation, thus providing additional energy to sustain cell proliferation and maintain the CSCs compartment. Notably, cisplatin exposure increases OCR dependency from glutamine oxidation but, more importantly, flexibility in usage of substrates other than glucose, long-chain fatty acids and glutamine to fuel mitochondrial respiration. Alternative energy sources could be found in short and medium chain fatty acids as well as other amino acids. This picture is even more complicated if we consider that in vivo CSCs can retrieve a wide range of catabolites from the microenvironment [45].

Cisplatin, which has been shown to enhance oxidative phosphorylation and glycolytic fluxes during glucose starvation, allows slow growing CSCs to direct the energy usage from cell division toward more efficient energy production, thus promoting cell survival. Increased expression levels of Sirt3 correlate with the cisplatin-induced metabolic reprogramming towards OxPhos in 3AB-OS CSCs. Several studies highlighted that oxidative phosphorylation enzymes are regulated via deacetylation by Sirt3, which plays a key role for mitochondrial energy production, metabolic homeostasis, and cell survival and longevity [46,47,48]. In addition, Sirt3 is known to potentiate ROS scavenging and antioxidant signaling, thereby reducing ROS-mediated cell damage and maintaining cellular homeostasis [49]. Notably, under starvation, Sirt3 coordinates the efficient use of available nutrients by promoting metabolic flexibility and funnel alternative sources into the TCA cycle [50]. Due to the lack of mitochondrial oxidative damage, it is conceivable that cisplatin acts mainly at a nuclear level in low glucose and, upon cisplatin-induced nuclear DNA damage, Sirt3 is transported from the nucleus to the mitochondria to exert its beneficial effects [36,51].

As glucose restriction did not augment CSCs sensitivity to cisplatin, we explored 3AB-OS metabolic adaptation during starvation for the search of alternative pathways to target, in order to potentiate cytotoxicity of glucose deprivation. Low glucose was shown to activate the autophagy flux as survival strategy. Autophagy activation is consistent with the observed upregulation of mitochondrial OxPhos during glucose deprivation, as autophagy has been reported to feed the TCA cycle and autophagy-deficient cells have showed less mitochondrial respiration under starvation [52]. Therefore, autophagy could contribute to enhance mitochondrial function through protein and organelles recycling.

## 5. Conclusions

Although cancer therapy has made great progress, osteosarcoma is still associated with poor prognosis in most patients. Moreover, current chemotherapeutic agents (a) cause severe side effects, ranging from malaise to the onset of metastatic tumors and toxicity to immune system, and (b) may result ineffective in killing CSCs. In this scenario, our work indicated that fine-tuning of nutrients or tailored manipulation of energy pathways may represent a complementary treatment to sensitize osteosarcoma cells to chemotherapy and kill CSCs, thus having the potential benefit to (a) limit drug-induced adverse effects through reduction of the effective drug concentration and (b) prevent tumor recurrence originating from CSCs. Indeed, an increasing body of evidence reports that nutrient supply modulation through diet improves tumor-bearing survival [53].

Comparative metabolic profiling of MG63 and 3AB-OS cell lines further proofs the existence of intratumor metabolic heterogeneity within osteosarcoma, with CSCs being more glycolytic while differentiated cells having high mitochondrial metabolism. These findings may be relevant to design a two-step strategy for complete eradication of cancer cells. During the first step, cisplatin treatment can be efficiently integrated with either glutamine deprivation or glutaminolysis inhibition to kill the bulk of the tumor cells (i.e., MG63 cells). During the second step, removal of residual CSCs (i.e., 3AB-OS cells) could be accomplished through time-controlled deprivation of glucose or inhibition of glycolysis, in combination with molecules targeting the autophagy pathway.

A limit of this study is that our findings are restricted to two osteosarcoma cell models and cannot be generalized. However, our work suggests a potential approach to lay bare metabolic addictions in primary tumor cell lines. Validation of our observations in other osteosarcoma cells will be the preliminary step towards future, in-depth investigations in animal models and patient tumor biopsies.

## Data Availability

Not applicable.
